# A case report: metastasis of melanoma to the heart in an era of immunotherapy

**DOI:** 10.1093/ehjcr/ytz182

**Published:** 2019-10-26

**Authors:** Christian B Poulsen, Kathrine S Weile, Henrik Schmidt, Steen H Poulsen

**Affiliations:** 1 Department of Cardiology, Regional Hospital West Jutland, Gl. Landevej 61, DK-7400 Herning, Denmark; 2 Department of Cardiology, Aarhus University Hospital, Palle Juul-Jensens Boulevard 99, DK-8200 Aarhus, Denmark; 3 Department of Medicine, Regional Hospital West Jutland, Gl. Landevej 61, DK-7400, Herning, Denmark; 4 Department of Oncology, Aarhus University Hospital, Palle Juul-Jensens Boulevard 99, DK-8200 Aarhus, Denmark

**Keywords:** Case report, Melanoma, Cardiac metastasis, Echocardiography

## Abstract

**Background:**

Cardiac metastasis of melanoma rarely causes heart failure symptoms and the recognition of cardiac involvement is in most cases first established post-mortem. Surgical removal might be considered in selected cases in patients with an inflow or outflow tract obstruction even though the survival remains poor. Frequently, the metastasis cannot be removed and therapeutic options include conventional chemotherapy or immunotherapy, which is currently recommended as first-line treatment. Since the introduction of immunotherapy survival in metastatic disease has significantly increased but data on patients treated for melanoma with cardiac involvement are scarce.

**Case summary:**

A 65-year-old man presented with dyspnoea and fatigue. Computed tomography scan revealed tumour processes in the heart, which was confirmed on echocardiography. Biopsies taken from fluorodeoxyglucose positron emission tomography positive lymph nodes in the axilla and groin showed melanoma. Analyses did not reveal BRAF mutation and the PD-L1 expression in tumour cells was below 1%. Treatment with ipilimumab and nivolumab was initiated and cardiopulmonary symptoms subsided during the following months with significant reduction in cardiac metastasis on echocardiography. Unfortunately, the patient developed immune checkpoint inhibitor-induced colitis and could no longer continue on the therapy. Due to development of extra-cardiac and cerebral metastasis, he was referred to palliative care.

**Discussion:**

This case demonstrates that timely treatment with immunotherapy could be a safe and effective option for melanoma with cardiac involvement. During treatment, the patient developed severe colitis, a known side effect to immunotherapy. Though this often can be managed with steroids it complicates further treatment.

**Learning points**

- Immunotherapy is effective in reducing cardiac metastasis of malignant melanoma without causing adverse cardiac events in the majority of patients.
- Therapy-induced colitis constitutes a significant clinical problem in this patient population.
- Melanoma patients with elevated troponin, N-terminal pro-B-type brain natriuretic peptide, or electrocardiogram changes should be referred for cardiac evaluation.

## Introduction

Melanoma carries a dismal prognosis with an increasing incidence reaching 19 cases per 100 000 in Northern Europe and thus constitutes a burden to public health.^1^ Cardiac metastasis has been documented in 47.2% of patients with melanoma post-mortem but in the majority of cases it was clinically silent.^2–4^ When cardiac involvement is diagnosed ante-mortem, symptoms are often related to tumour location and varies from functional dyspnoea, chest pain to dizziness, or syncope.^5^ Since the introduction of immunotherapy in melanoma management, survival has improved significantly for metastatic disease however when cardiac involvement is present data are lacking in regard to prognosis and response to treatment.^6^

## Timeline

**Table:** 

Time	Event	Findings
August 2018	Fatigue and dyspnoea.	
September 3	Clinical evaluation by general practitioner.	
September 27	Computed tomography (CT) scan of chest and abdomen.	Tumour infiltration in left ventricle and multiple enlarged lymph nodes in the mediastinum and retro peritoneum.
October 2	Hospital consultation and echocardiography (*Figure 1C*).	Tumour in the right ventricle and ventricular septum.
October 8	Fluorodeoxyglucose positron emission tomography scan (*Figure 1D*).	Multiple fluorodeoxyglucose positive masses in the heart, right lung, and lymph nodes in the groin.
October 9	Biopsy from lymph nodes.	
October 13	Pathology shows malignant melanoma.	
October 18	Cardiac evaluation and echocardiography (*Figures 1E* and *2A–C*). Blurred vision, referred to cerebral magnetic resonance imaging (MRI). Commences prednisolone treatment.	Multiple cardiac metastases were found on contrast echocardiography. Cerebral MRI shows metastasis in left occipital and temporal lope.
October 24	Pathology shows no BRAF mutation or PD-L1 expression.	
October 25	Cardiac MRI (*Figure 1F*).	Extensive metastatic infiltration in the left ventricle.
November 1	First treatment with ipilimumab 78 mg and nivolumab 235 mg.	
November 23	Second treatment with ipilimumab 78 mg and nivolumab 235 mg.	
December 14	Third treatment with ipilimumab 78 mg and nivolumab 235 mg.	
December 17	Cardiac evaluation with echocardiography (*Figure 2D–F*).	Regression in metastatic size.
December 20	Diarrhoea.	
January 7, 2019	Persistent diarrhoea, commences infliximab.	
January 29	Routine cerebral MRI and CT scan of the chest and abdomen after commencing immunotherapy. Echocardiography (*Figure 3B* and *C*).	Progression of metastasis on cerebral MRI. Computed tomography scan reveals progression of extra-cardiac metastasis. Echocardiography shows persistent reduction in cardiac metastasis and improvement in global longitudinal strain score.
February 1	Referred to palliative care.	

## Case presentation 

**Figure 1:**
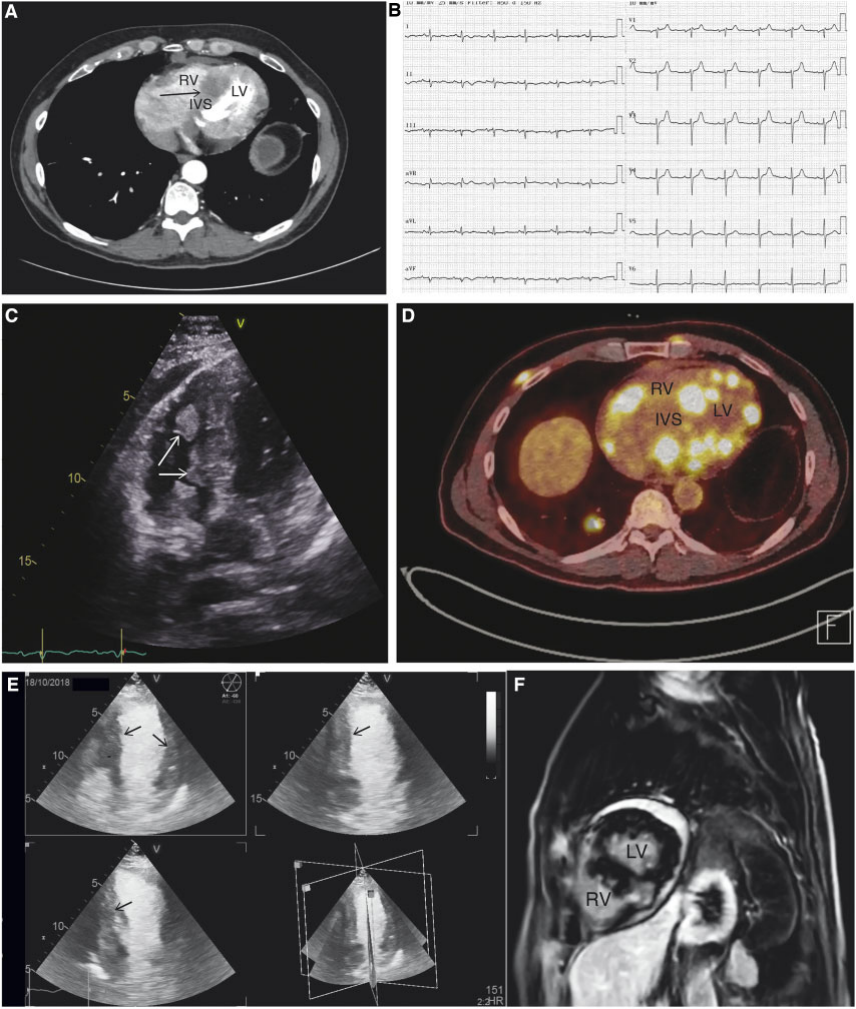
(*A*) Computed tomography scan of the chest showing an infiltrating tumour (arrow) in the interventricular septum. IVS, interventricular septum; LV, left ventricle; RV, right ventricle. (*B*) Electrocardiogram with low voltage and inverted T waves in inferior leads (II, III, and aVF). (*C*) Echocardiogram showing metastasis in the interventricular septum and right ventricle (arrows). (*D*) Fluorodeoxyglucose positron emission tomography combined with computed tomography in the same region as shown in *A* in addition to the tumour located in the interventricular septum additional metastatic processes are visible in the myocardium of both right and left ventricles. (*E*) Contrast echocardiography showing multiple metastasis in the septum and left ventricle (arrows). (*F*) Cardiac magnetic resonance imaging showing extensive melanoma infiltration in left ventricle (T1 weighted sequence).

A 65-year-old Caucasian male was referred from his general practitioner after complaining of fatigue and shortness of breath at moderate exertion for 3–5 weeks. He had no prior medical history and did not use any prescribed medication. A computed tomography (CT) scan of the chest and abdomen revealed multiple enlarged lymph nodes in the mediastinum and retro peritoneum. A large infiltrating tumour was found in the left ventricle along with a small pericardial effusion (*Figure 1A*) and sub-segmental pulmonary atelectasis.

At physical examination, heart and lung auscultations were unremarkable and no signs of ascites or peripheral oedema were present. No cutaneous lesions were found though multiple hard subcutaneous masses were felt on palpation of the chest and abdomen. Saturation was 96% while breathing ambient air. Blood pressure was 123/85 mmHg and heart rate 100 b.p.m. The electrocardiogram (ECG) showed sinus rhythm with low voltage and T-wave inversion in the inferior leads suggestive of myocardial involvement (*Figure 1B*).^7^ Echocardiogram revealed masses in the right ventricle and ventricular septum (*Figure 1C*), with normal left ventricular ejection fraction >60% and preserved diastolic function. A positron emission tomography (PET) scan revealed multiple fluorodeoxyglucose (FDG) positive masses in the heart (*Figure 1D*), right lung, and lymph nodes in the thorax and groin. Consistent with the FDG-PET scan multiple cardiac metastases were found on contrast echocardiography (*Figure 1E*) and extensive myocardial infiltration was present on cardiac magnetic resonance imaging (MRI, *Figure 1F*). No involvement of the cardiac valves was found on echocardiography, cardiac MRI, or FDG-PET.

**Figure 2:**
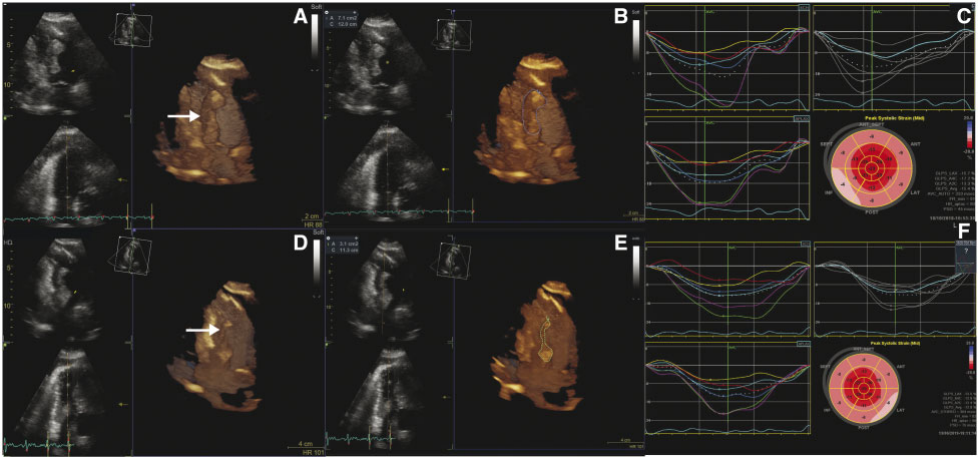
(*A*) Echocardiography before treatment with ipilimumab and nivolumab shows tumour in the right ventricle (arrow) with traced area of 7.1 cm^2^ (*B*). (*C*) Global longitudinal strain was reduced with apical sparing. (*D*) Echocardiography following third treatment with ipilimumab and nivolumab (6 weeks after treatment initiation) shows substantial regression in tumour size (arrow) with traced area reduced to 3.1 cm^2^ (*E*). No improvement in global longitudinal strain was noted at this time (*F*).

Biopsies taken from FDG positive lymph nodes showed malignant melanoma and subsequent analysis did not reveal BRAF mutation or PD-L1 expression. Shortly after, the patient developed blurred vision and a cerebral MRI revealed metastasis in the left occipital and temporal lobe. To manage symptoms, the patient commenced treatment with prednisolone 50 mg once daily and was subsequently reduced to 12.5 mg daily during the following weeks. Due to the patient’s metastatic disease with cerebral involvement, he was referred for combination therapy with ipilimumab 78 mg (anti-CTLA 4 antibody) and nivolumab 235 mg (anti-PD-1 antibody) every third week while on steroid treatment.^8^ Cardiac evaluation before ipilimumab and nivolumab treatment showed progression of metastasis in the right ventricle (*Figure 2A* and *B*). Strain analysis revealed reduced global longitudinal strain (GLS) to −15.4% on average and apical sparing consistent with infiltrative disease (*Figure 2C*).^9^ Laboratory studies showed elevated N-terminal pro-B-type brain natriuretic peptide (NT-pro-BNP) 1391 ng/L (<300 ng/L) and troponin T, 21 ng/L (<14 ng/L). At the time of second treatment, NT-pro-BNP and troponin T increased to 3774 ng/L and 62 ng/L, respectively, suggestive of myocardial injury secondary to immunotherapy. After completing the third treatment, the patient felt a complete relief of his initial symptoms of dyspnoea and fatigue. Clinical evaluation showed reduction in subcutaneous metastasis and the ECG revealed sinus rhythm with normalization of T waves in the inferior leads. Subsequent echocardiography confirmed reduction in the size of cardiac metastasis (*Figure 2D* and *E*) however GLS score did not improve (−12.9%) on average (*Figure 2F*). Laboratory studies showed reduced NT-pro-BNP 1074 ng/L and TnT 20 ng/L. Following cardiac evaluation, the patient developed severe colitis for which immunotherapy was discontinued and his gastrointestinal symptoms managed by one treatment of TNF-alpha antibody (infliximab 5 mg/kg) and methylprednisolone 80 mg daily for 1 week. The patient subsequently resumed oral steroid treatment initially with prednisolone 75 mg once daily and was gradually reduced during the following weeks to 25 mg daily.

**Figure 3:**
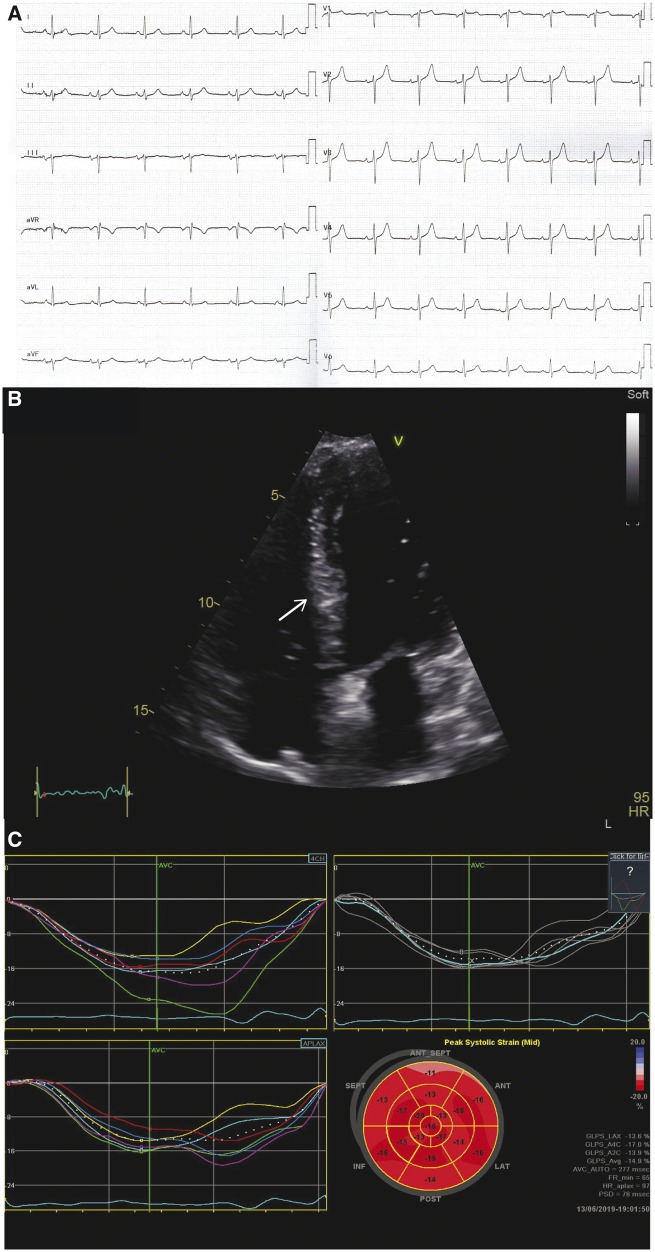
(*A*) Electrocardiogram obtained 12 weeks after treatment initiation with normalization of T waves in inferior leads (II, III, and aVF). (*B*) Echocardiogram obtained 12 weeks after treatment initiation showing persistent reduction in cardiac metastatic size (arrows) with improvement in global longitudinal strain and normalization of contraction pattern (*C*).

A routine MRI scan performed 3 months after treatment initiation revealed progression of cerebral metastasis and CT scan of the thorax and abdomen showed increase in extra-cardiac metastasis, a small peripheral pulmonary embolus, and no pleural effusions. Clinical evaluation revealed unremarkable ECG (*Figure 3A*), persistent reduction in cardiac metastasis on echocardiography (*Figure 3B*) and a notable improvement in GLS score to −14.9% on average with normalization of contraction pattern and Troponin I < 10 ng/L. No signs of increased pulmonary pressure was observed. Pulmonary embolism was treated with weight-adjusted low-molecular heparin daily. Due to deteriorating cognitive function and progression of extra-cardiac metastasis prednisolone dose was increased to 75 mg daily and the patient was referred to palliative care.

## Discussion

We present a case of symptomatic cardiac melanoma, treated with immune checkpoint inhibitors resulting in regression of cardiopulmonary symptoms and reduction in cardiac metastatic size, which persisted 6 weeks after discontinuation of therapy. To our knowledge, this is the first report of cardiac melanoma treated with both ipilimumab and nivolumab.

A decade ago metastatic melanoma had a 5-year survival rate of 5–10% on standard chemotherapies but with the introduction of immunotherapies in 2011 survival rates have been increasing.^10^^,^^11^ Nivolumab has significantly prolonged overall survival compared with conventional chemotherapy (dacarbazine)^12^ and in CheckMate O67 treatment with ipilimumab and nivolumab prolonged survival compared with ipilimumab after 4 years of follow-up.^13^

When cardiac melanoma is present, the most common location is in relation to the right-sided heart chambers.^14^ Though melanoma in the heart has been observed in up to 47.2% of patients post-mortem, finding patients presenting with symptoms primarily relating to the cardiovascular system is rare.^2^^,^^3^ Patients without cardiac symptoms should therefore be offered a screening of cardiac involvement by ECG, troponins, and NT-pro-BNP and in cases of abnormalities a two- and three-dimensional echocardiography should be performed to identify conditions such as; outflow tract obstruction, pericardial effusion, or signs of pulmonary embolism. In symptomatic cases with significant obstructive tumour masses cardiac surgery might be an option, but even with early surgical intervention prognosis remain poor mainly due to diffuse myocardial infiltration and metastasis in other organ systems.^4^ Though the patient had extra-cardiac metastasis that progressed during immunotherapy, this case demonstrates that treatment with ipilimumab and nivolumab vary rarely affects cardiac function and provides clinical benefits in patients with cardiac melanoma.

With the increased use of immune checkpoint inhibitors, toxicity is increasingly recognized as a clinical problem. For patients treated with ipilimumab and nivolumab, the most common symptoms are gastrointestinal. Particular diarrhoea which occurs in up to 30% of patients in clinical trials treated with ipilimumab.^15^ The patient discussed did develop immune checkpoint inhibitor-induced colitis and his symptoms were managed by administration of prednisolone and TNF-alpha antibody (infliximab). However, due to the severity of the colitis, the patient was not able to continue immunotherapy. A rare but serious toxic effect is immune-mediated myocarditis, which has been reported in 0.27% of patients treated with ipilimumab and nivolumab.^16^ This diagnosis should be considered if patients present with clinical deterioration in the weeks following treatment initiation.

Brain metastases are common in patients with melanoma and are found in more than 75% of cases at the time of death.^17^ Historically prognosis has been poor with a median overall survival of 2–5 months and only 5% surviving in the long-term (>5 years). Due to its poor prognosis randomized phase III trials are warranted but data from a randomized phase II study suggest that this population might benefit from combination therapy as well.^18^^,^^19^ In the current case, the patient initially reported regression of cerebral symptoms but had increased cognitive problems and progression of cerebral metastasis on magnetic resonance while on combination therapy.

## Conclusion

Despite cerebral progression of melanoma, this case demonstrates clinical benefits of treating cardiac melanoma with ipilimumab and nivolumab causing regression of cardiopulmonary symptoms and metastatic size.

## Lead author biography

**Figure:**
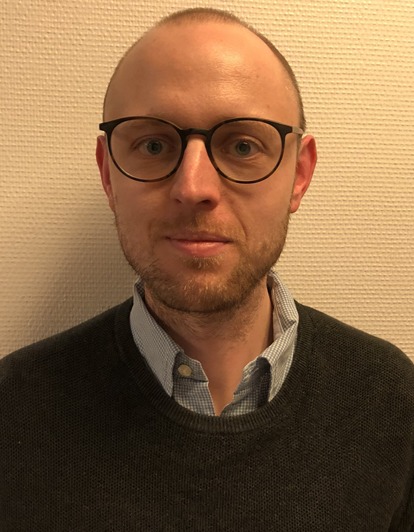


Christian B. Poulsen is a senior registrar in cardiology at the Regional Hospital West Jutland in Herning, Denmark. He has previously performed experimental studies of atherosclerosis in porcine models as part of his PhD.

## Supplementary material

Supplementary material is available at *European Heart Journal - Case Reports* online.

**Slide sets:** A fully edited slide set detailing this case and suitable for local presentation is available online as Supplementary data.

**Consent:** The author/s confirm that written consent for submission and publication of this case report including image(s) and associated text has been obtained from the patient in line with COPE guidance.

**Conflict of interest:** none declared.

## Supplementary Material

ytz182_Supplementary_Slide_Set
