# Supplementary material for: A case report: metastasis of melanoma to the heart in an era of immunotherapy
Source: Eur Heart J Case Rep. 2019 Oct 26;3(4):1–7. doi: 10.1093/ehjcr/ytz182 (PMC7042148; doi:10.1093/ehjcr/ytz182)
Supplement: ytz182_Supplementary_Slide_Set [file ytz182_supplementary_slide_set.pptx]

## Slide 1
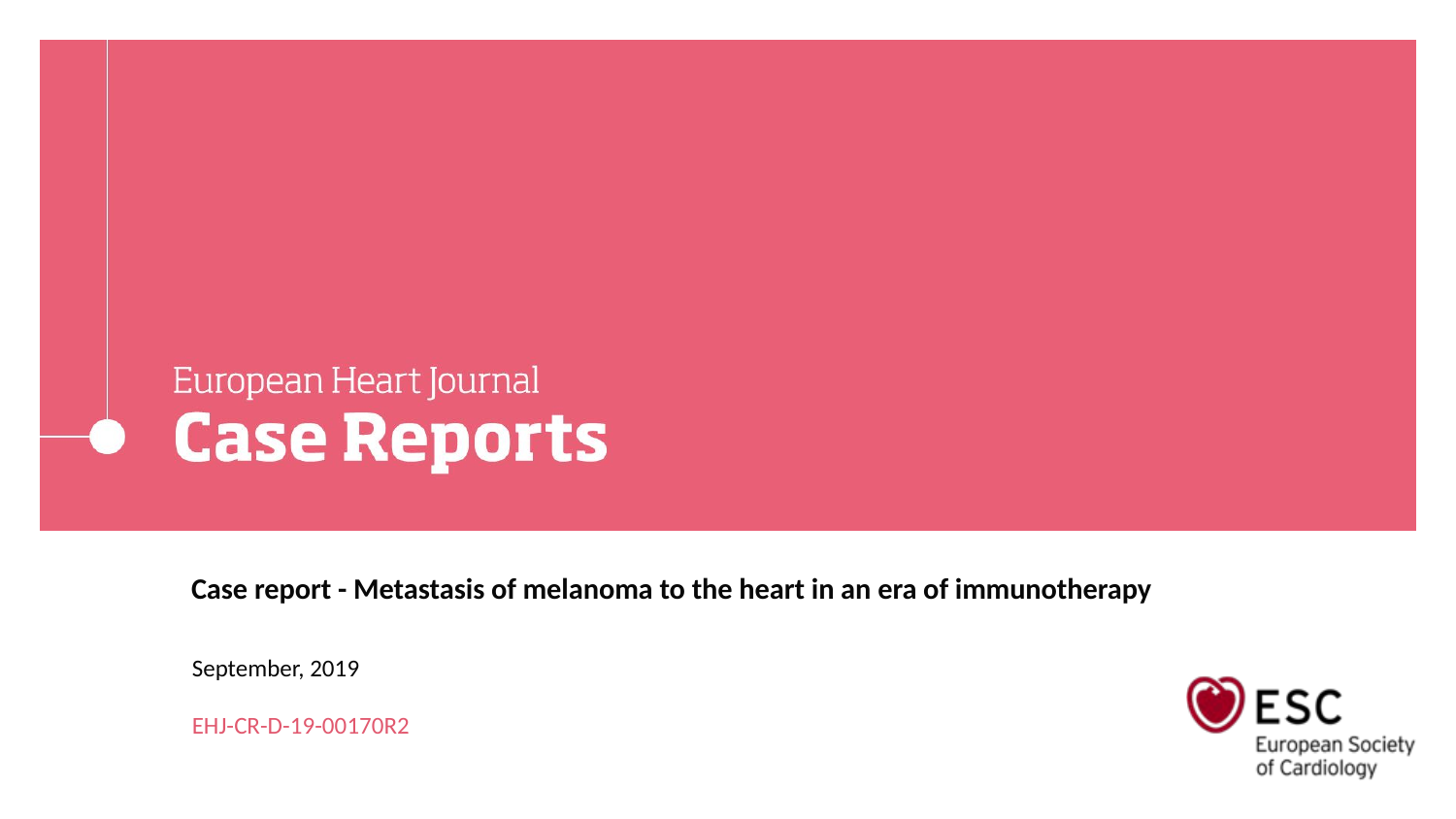

# Case report - Metastasis of melanoma to the heart in an era of immunotherapy
September, 2019
EHJ-CR-D-19-00170R2

## Slide 2
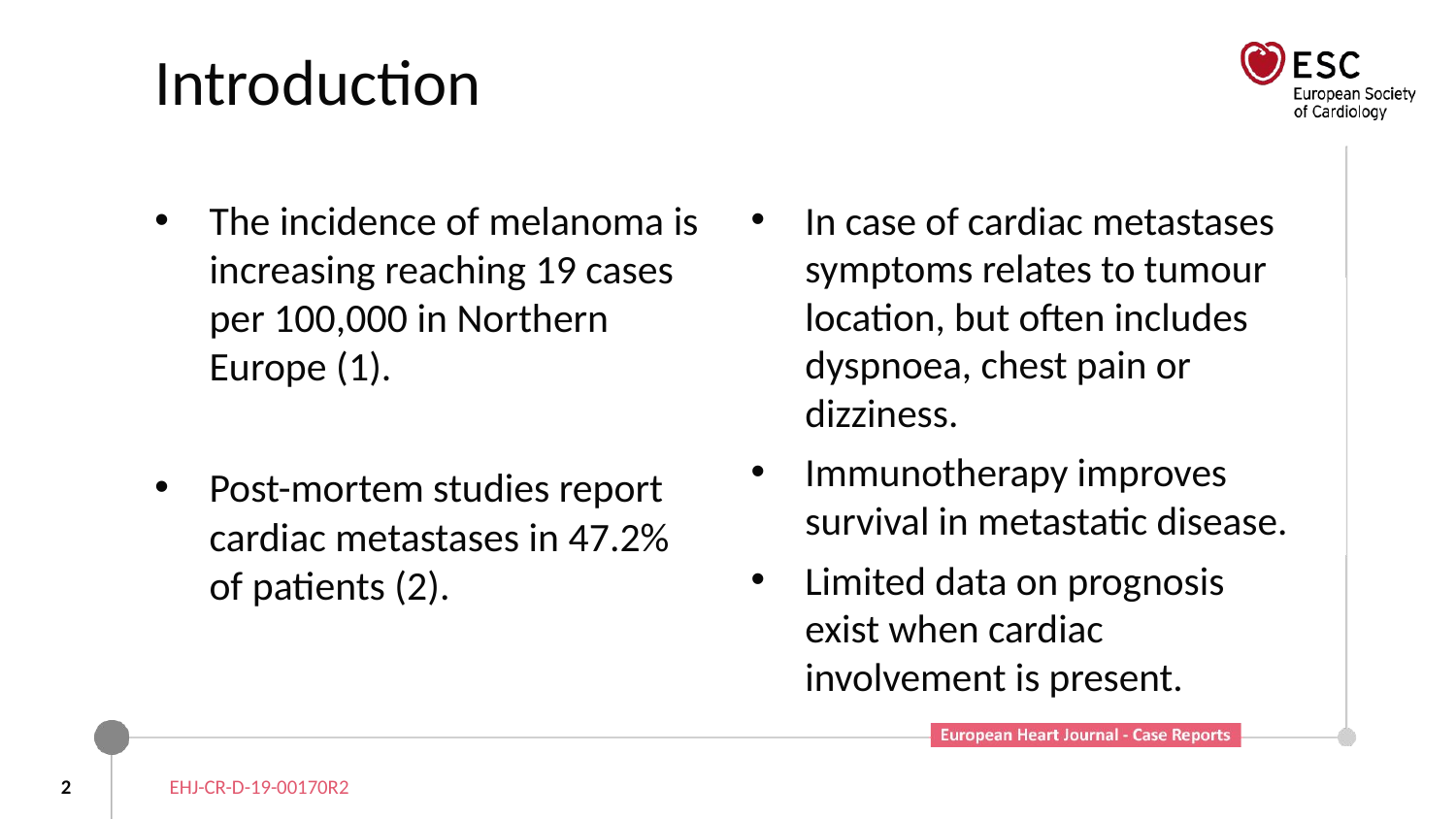

# Introduction
The incidence of melanoma is increasing reaching 19 cases per 100,000 in Northern Europe (1).
Post-mortem studies report cardiac metastases in 47.2% of patients (2).
In case of cardiac metastases symptoms relates to tumour location, but often includes dyspnoea, chest pain or dizziness.
Immunotherapy improves survival in metastatic disease.
Limited data on prognosis exist when cardiac involvement is present.
2
EHJ-CR-D-19-00170R2

## Slide 3
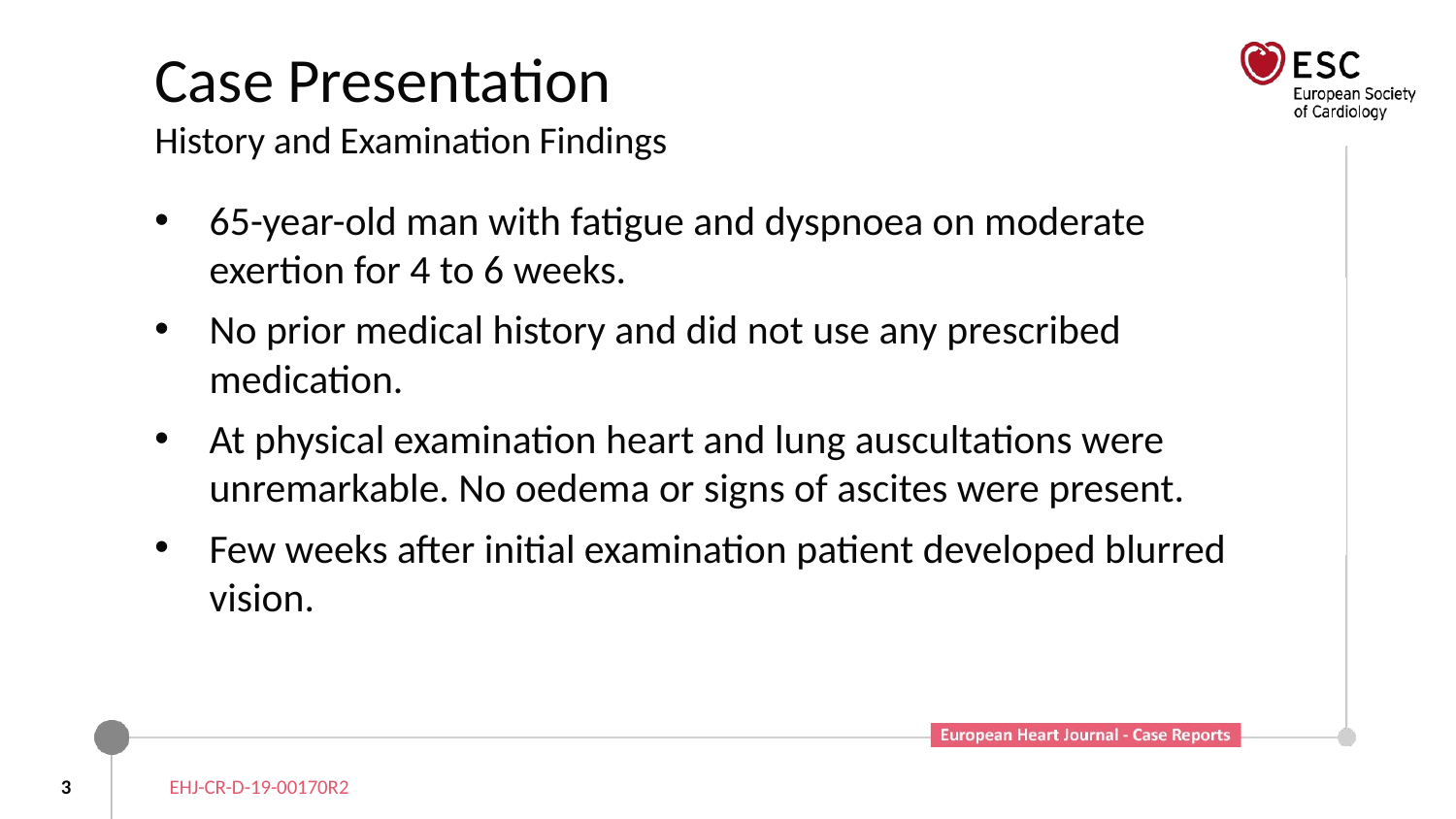

# Case Presentation History and Examination Findings
65-year-old man with fatigue and dyspnoea on moderate exertion for 4 to 6 weeks.
No prior medical history and did not use any prescribed medication.
At physical examination heart and lung auscultations were unremarkable. No oedema or signs of ascites were present.
Few weeks after initial examination patient developed blurred vision.
3
EHJ-CR-D-19-00170R2

## Slide 4
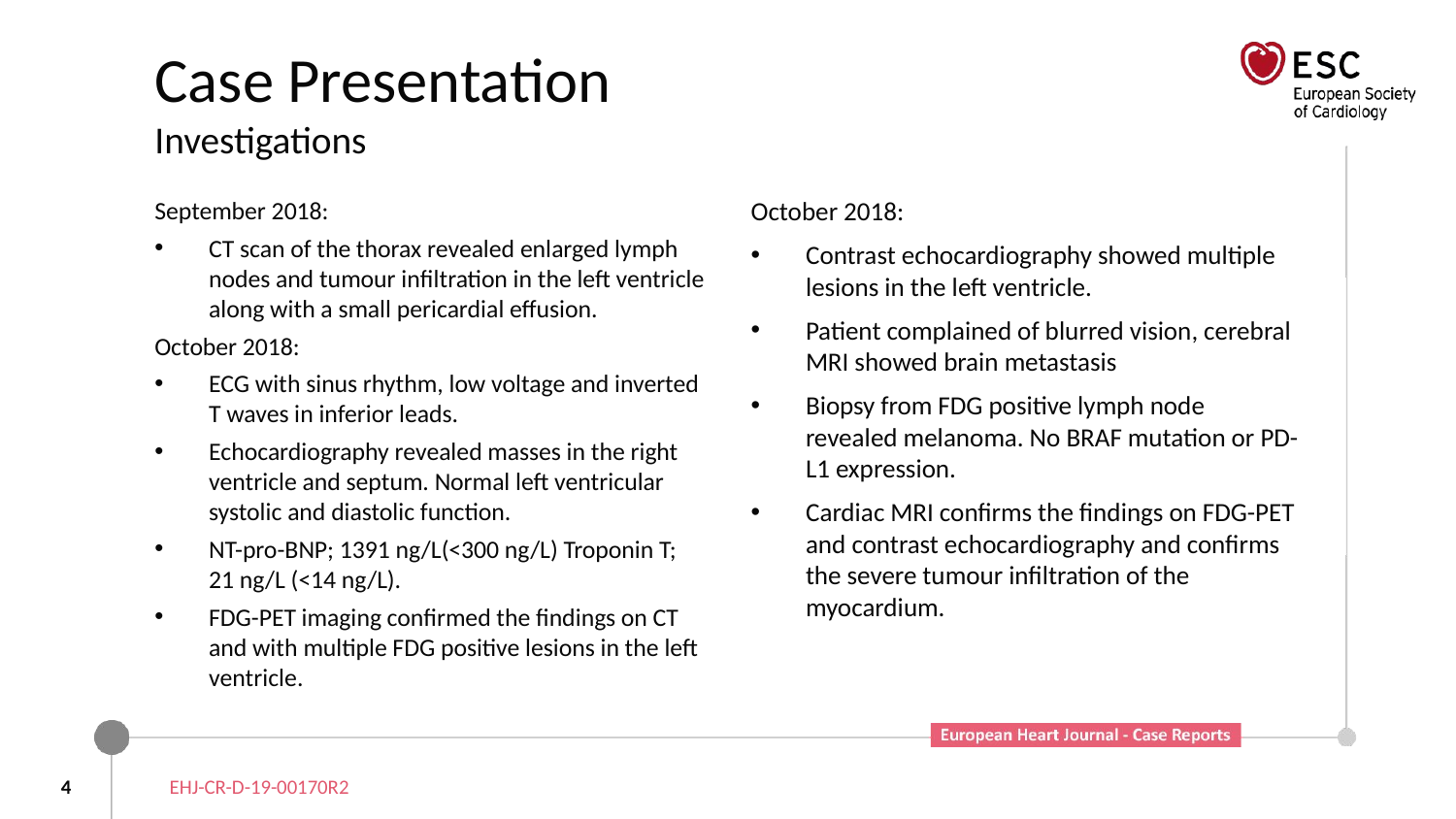

# Case Presentation Investigations
September 2018:
CT scan of the thorax revealed enlarged lymph nodes and tumour infiltration in the left ventricle along with a small pericardial effusion.
October 2018:
ECG with sinus rhythm, low voltage and inverted T waves in inferior leads.
Echocardiography revealed masses in the right ventricle and septum. Normal left ventricular systolic and diastolic function.
NT-pro-BNP; 1391 ng/L(<300 ng/L) Troponin T; 21 ng/L (<14 ng/L).
FDG-PET imaging confirmed the findings on CT and with multiple FDG positive lesions in the left ventricle.
October 2018:
Contrast echocardiography showed multiple lesions in the left ventricle.
Patient complained of blurred vision, cerebral MRI showed brain metastasis
Biopsy from FDG positive lymph node revealed melanoma. No BRAF mutation or PD-L1 expression.
Cardiac MRI confirms the findings on FDG-PET and contrast echocardiography and confirms the severe tumour infiltration of the myocardium.
4
EHJ-CR-D-19-00170R2

## Slide 5
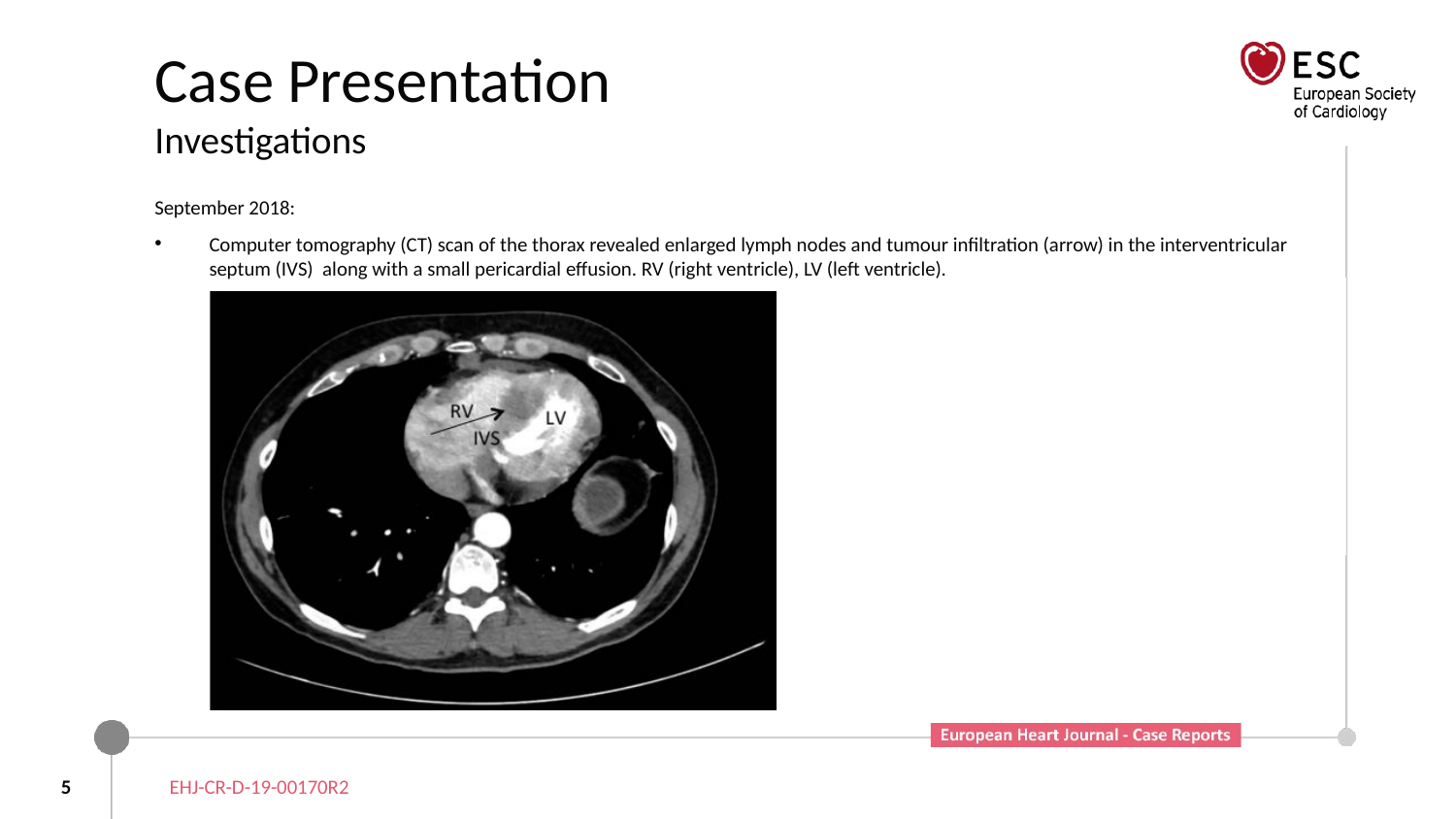

# Case Presentation Investigations
September 2018:
Computer tomography (CT) scan of the thorax revealed enlarged lymph nodes and tumour infiltration (arrow) in the interventricular septum (IVS) along with a small pericardial effusion. RV (right ventricle), LV (left ventricle).
5
EHJ-CR-D-19-00170R2

## Slide 6
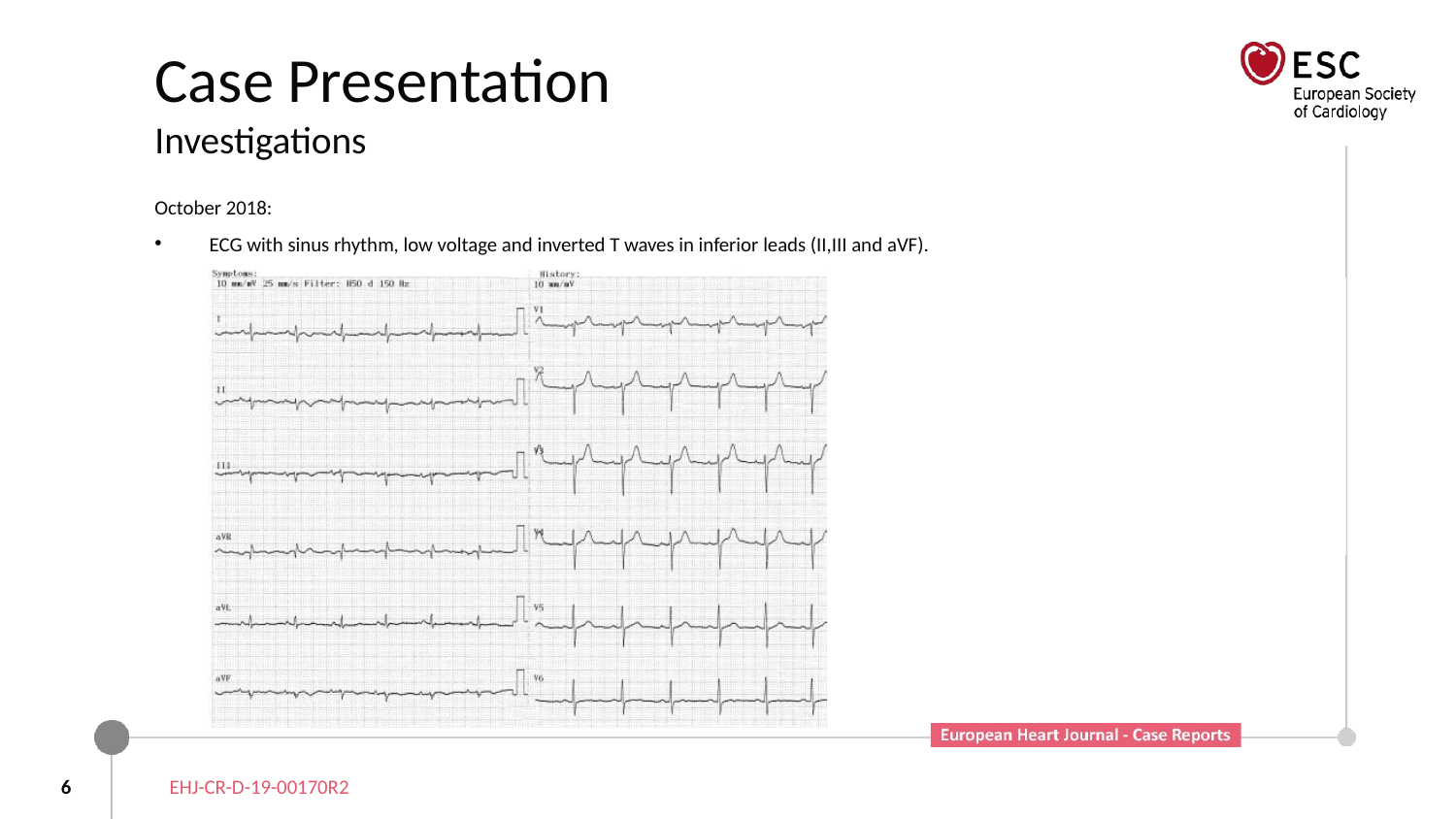

# Case Presentation Investigations
October 2018:
ECG with sinus rhythm, low voltage and inverted T waves in inferior leads (II,III and aVF).
6
EHJ-CR-D-19-00170R2

## Slide 7
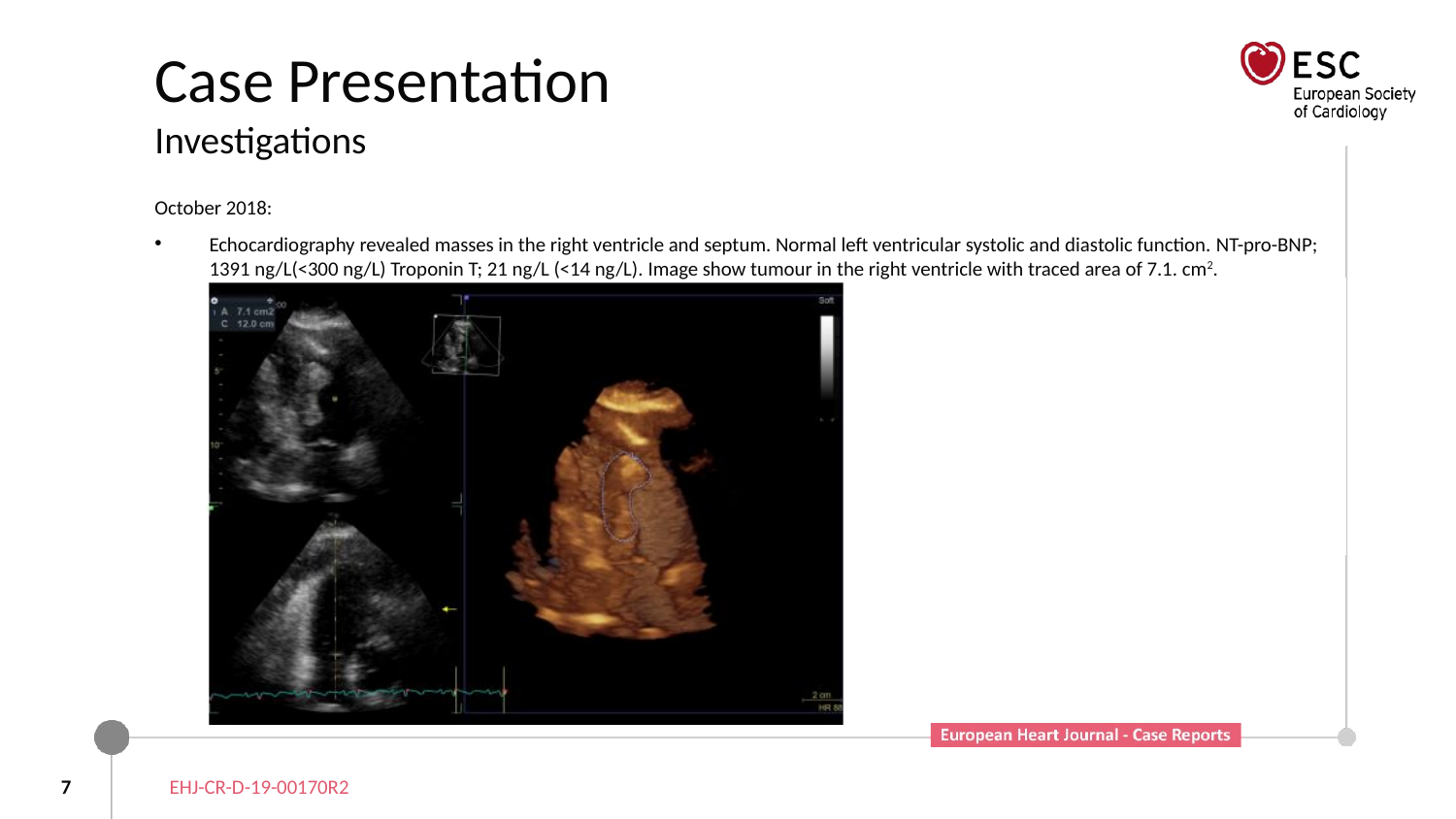

# Case Presentation Investigations
October 2018:
Echocardiography revealed masses in the right ventricle and septum. Normal left ventricular systolic and diastolic function. NT-pro-BNP; 1391 ng/L(<300 ng/L) Troponin T; 21 ng/L (<14 ng/L). Image show tumour in the right ventricle with traced area of 7.1. cm2.
7
EHJ-CR-D-19-00170R2

## Slide 8
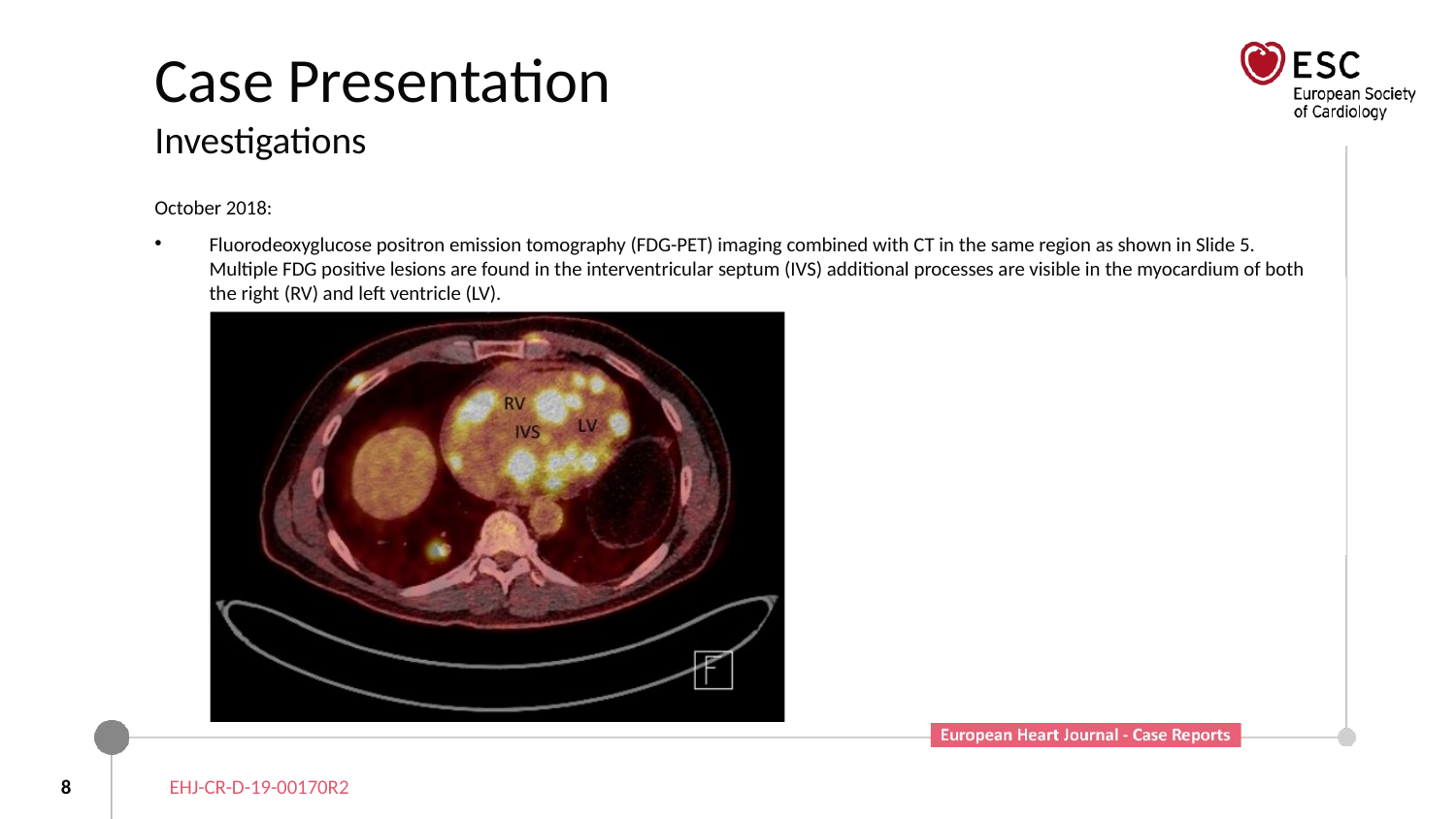

# Case Presentation Investigations
October 2018:
Fluorodeoxyglucose positron emission tomography (FDG-PET) imaging combined with CT in the same region as shown in Slide 5. Multiple FDG positive lesions are found in the interventricular septum (IVS) additional processes are visible in the myocardium of both the right (RV) and left ventricle (LV).
8
EHJ-CR-D-19-00170R2

## Slide 9
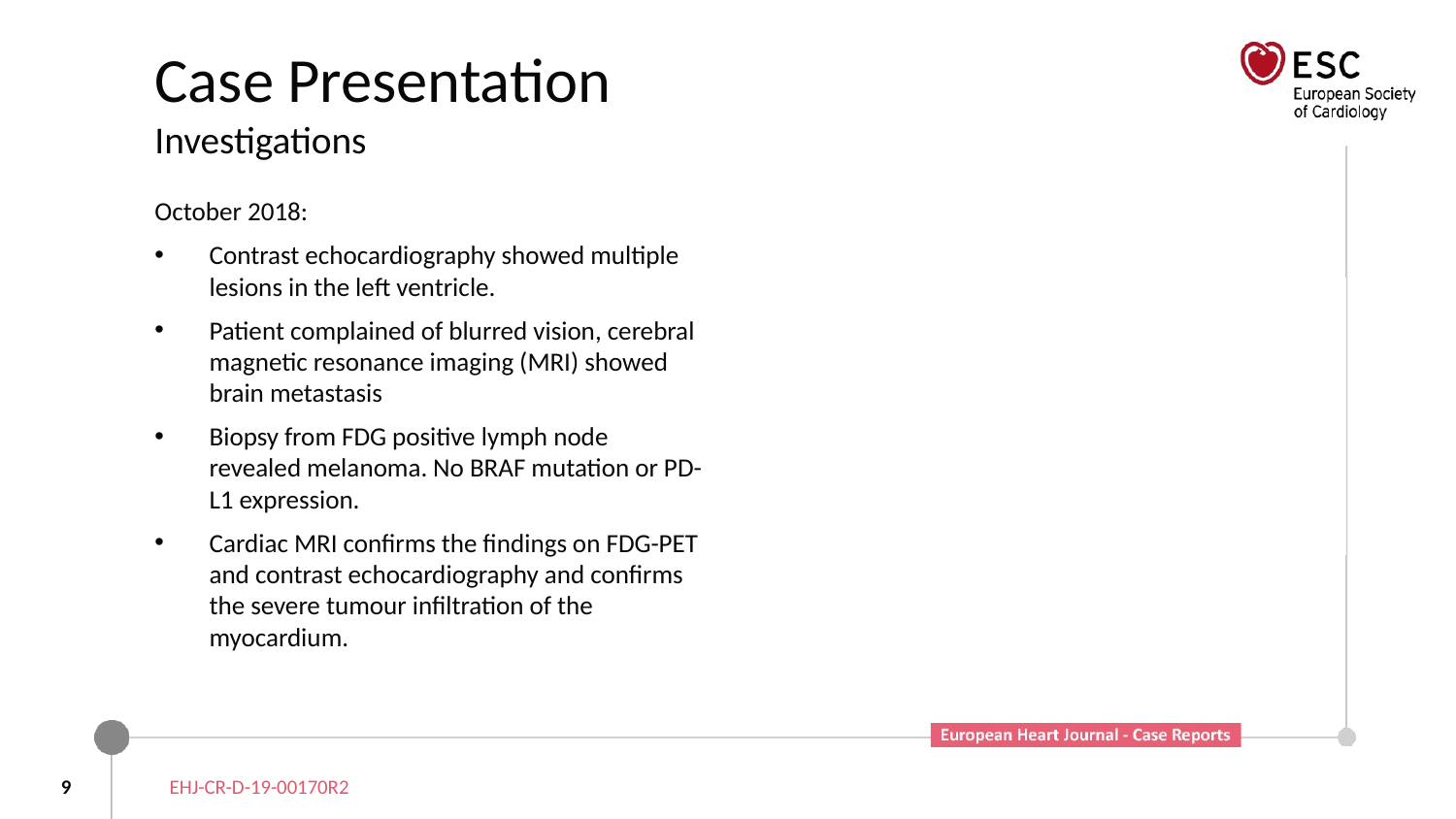

# Case Presentation Investigations
October 2018:
Contrast echocardiography showed multiple lesions in the left ventricle.
Patient complained of blurred vision, cerebral magnetic resonance imaging (MRI) showed brain metastasis
Biopsy from FDG positive lymph node revealed melanoma. No BRAF mutation or PD-L1 expression.
Cardiac MRI confirms the findings on FDG-PET and contrast echocardiography and confirms the severe tumour infiltration of the myocardium.
9
EHJ-CR-D-19-00170R2

## Slide 10
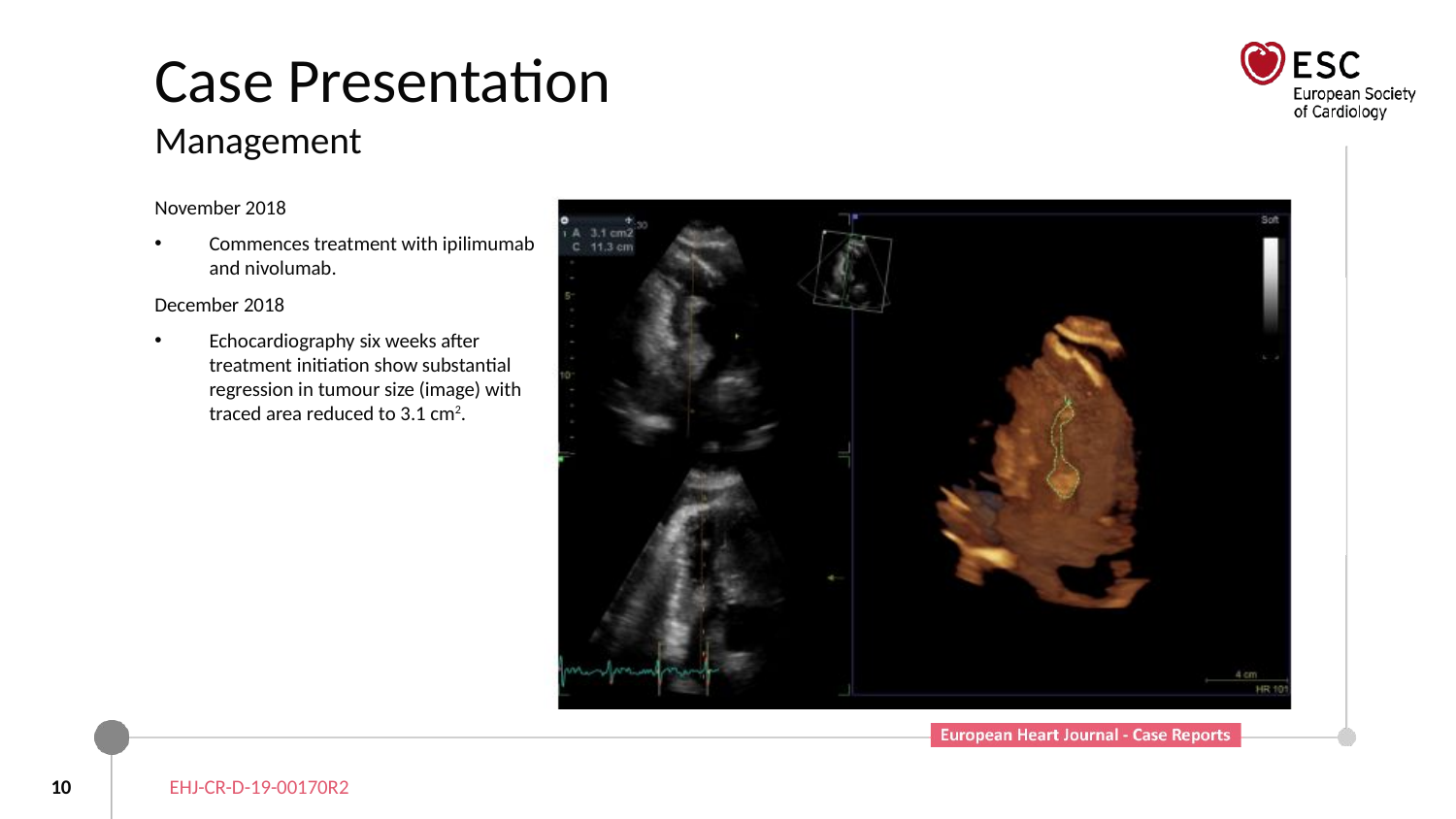

# Case Presentation Management
November 2018
Commences treatment with ipilimumab and nivolumab.
December 2018
Echocardiography six weeks after treatment initiation show substantial regression in tumour size (image) with traced area reduced to 3.1 cm2.
10
EHJ-CR-D-19-00170R2

## Slide 11
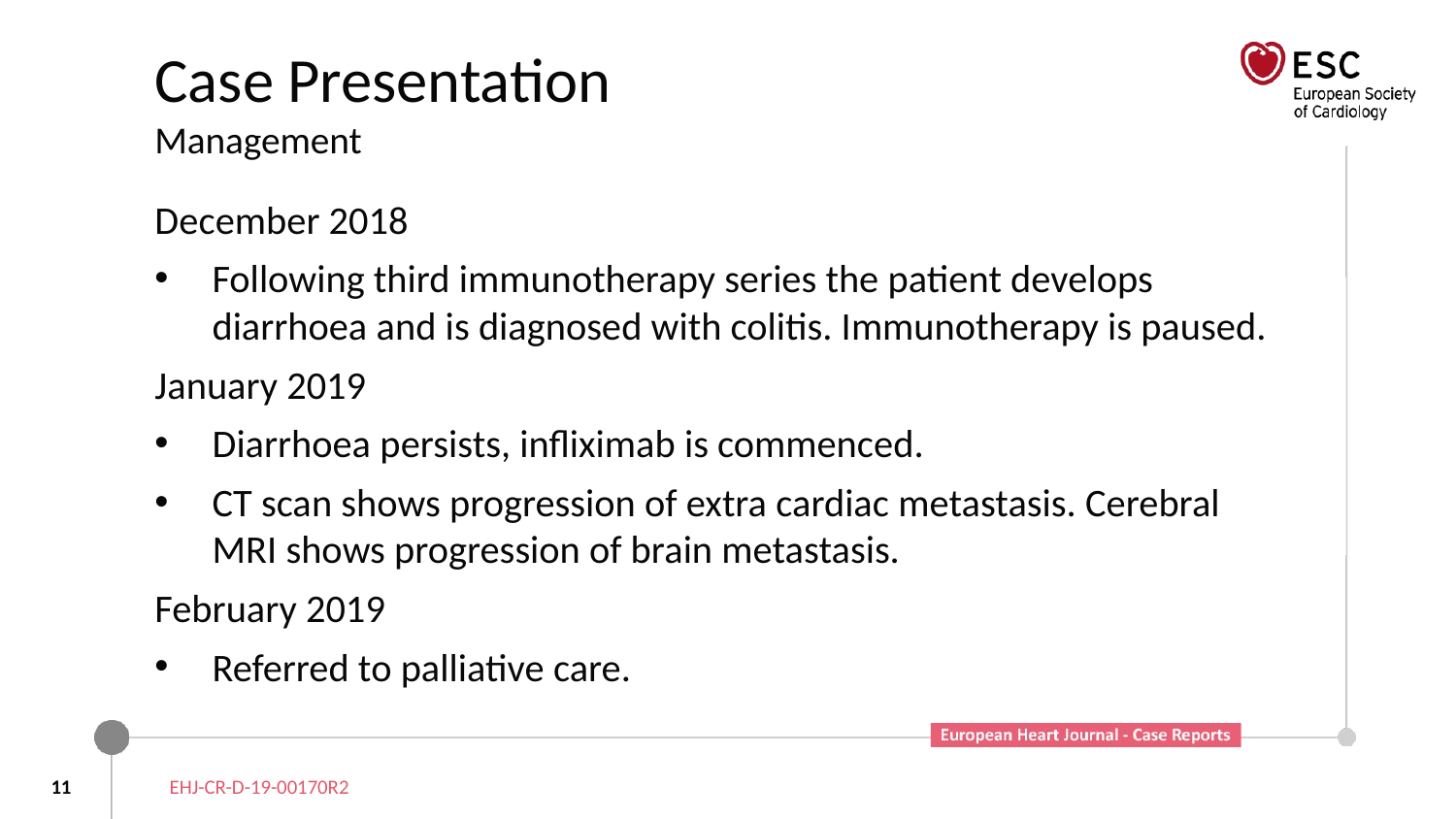

# Case Presentation Management
December 2018
Following third immunotherapy series the patient develops diarrhoea and is diagnosed with colitis. Immunotherapy is paused.
January 2019
Diarrhoea persists, infliximab is commenced.
CT scan shows progression of extra cardiac metastasis. Cerebral MRI shows progression of brain metastasis.
February 2019
Referred to palliative care.
11
EHJ-CR-D-19-00170R2

## Slide 12
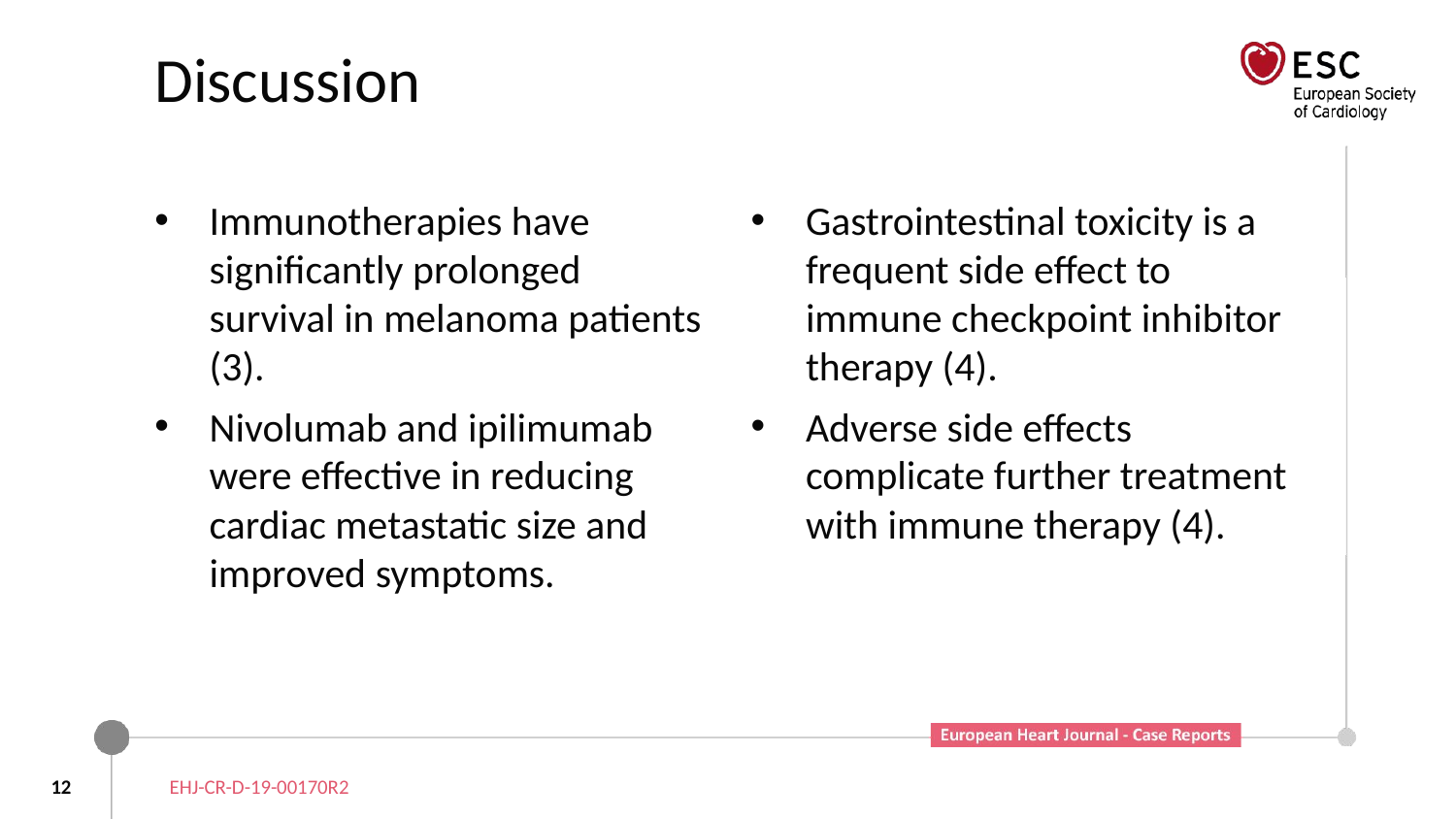

# Discussion
Immunotherapies have significantly prolonged survival in melanoma patients (3).
Nivolumab and ipilimumab were effective in reducing cardiac metastatic size and improved symptoms.
Gastrointestinal toxicity is a frequent side effect to immune checkpoint inhibitor therapy (4).
Adverse side effects complicate further treatment with immune therapy (4).
12
EHJ-CR-D-19-00170R2

## Slide 13
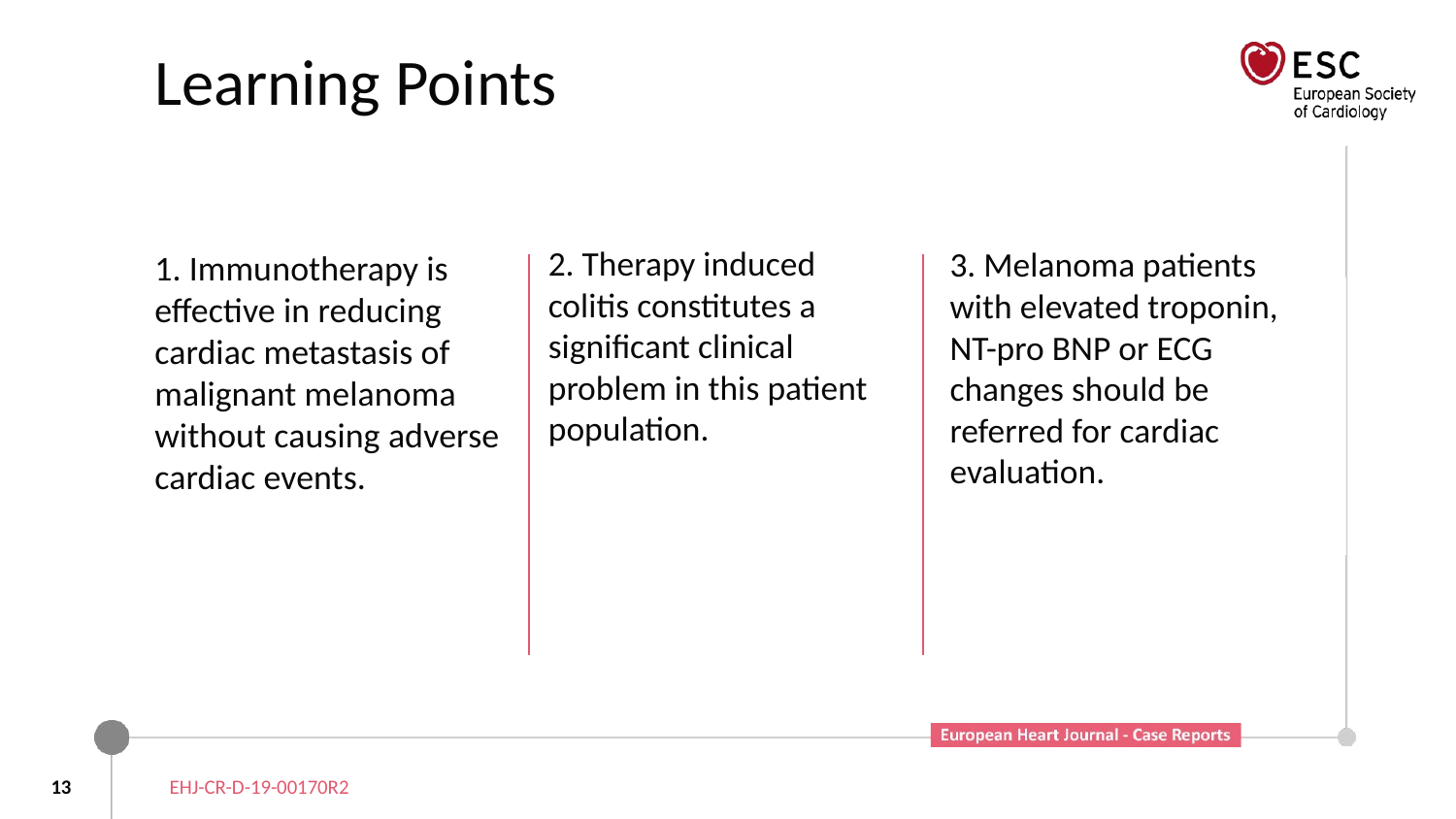

# Learning Points
2. Therapy induced colitis constitutes a significant clinical problem in this patient population.
3. Melanoma patients with elevated troponin, NT-pro BNP or ECG changes should be referred for cardiac evaluation.
1. Immunotherapy is effective in reducing cardiac metastasis of malignant melanoma without causing adverse cardiac events.
13
EHJ-CR-D-19-00170R2

## Slide 14
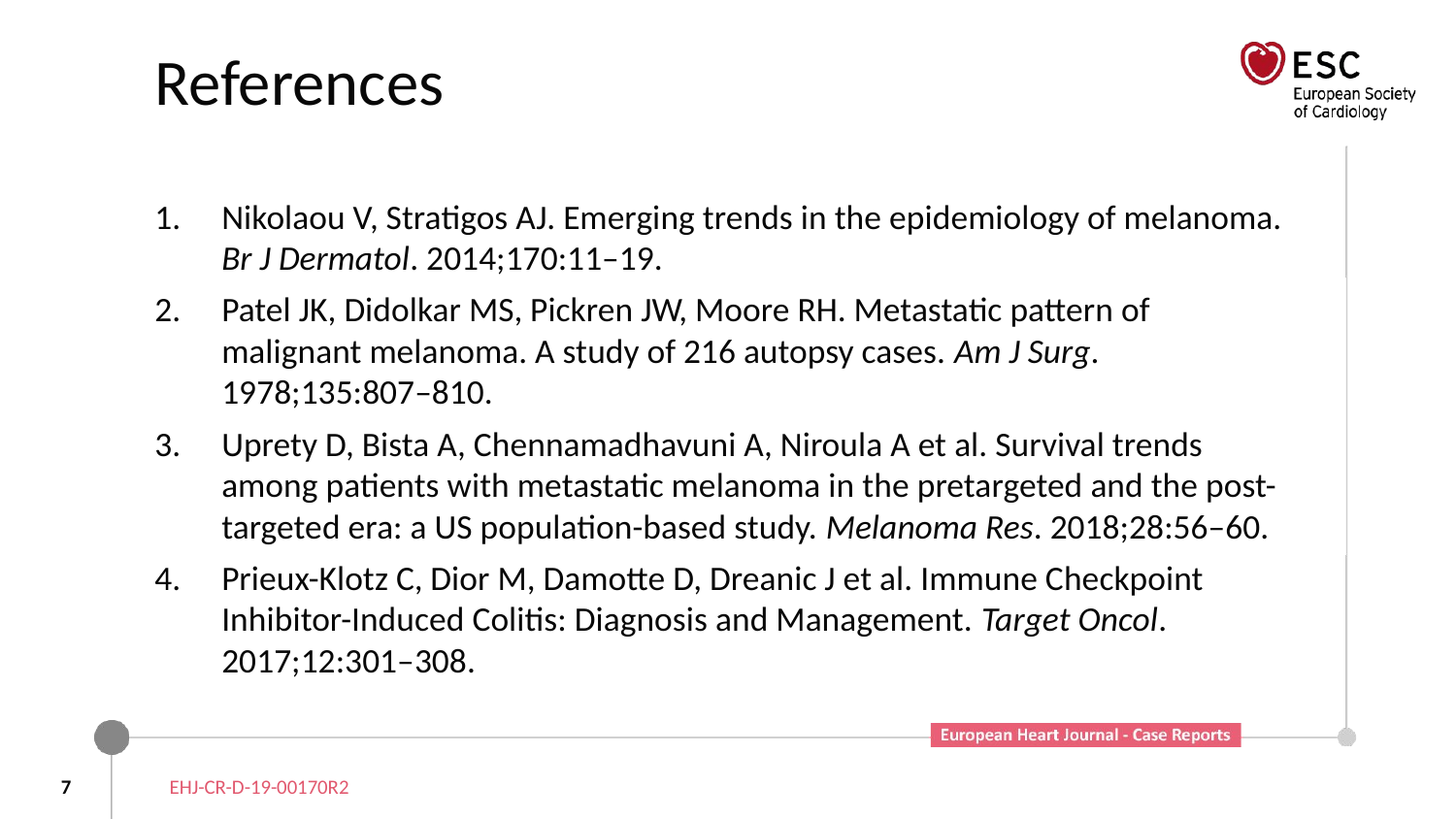

# References
Nikolaou V, Stratigos AJ. Emerging trends in the epidemiology of melanoma. Br J Dermatol. 2014;170:11–19.
Patel JK, Didolkar MS, Pickren JW, Moore RH. Metastatic pattern of malignant melanoma. A study of 216 autopsy cases. Am J Surg. 1978;135:807–810.
Uprety D, Bista A, Chennamadhavuni A, Niroula A et al. Survival trends among patients with metastatic melanoma in the pretargeted and the post-targeted era: a US population-based study. Melanoma Res. 2018;28:56–60.
Prieux-Klotz C, Dior M, Damotte D, Dreanic J et al. Immune Checkpoint Inhibitor-Induced Colitis: Diagnosis and Management. Target Oncol. 2017;12:301–308.
7
EHJ-CR-D-19-00170R2
